# Corrigendum to “Leucine–glycine and carnosine dipeptides prevent diabetes induced by multiple low‐doses of streptozotocin in an experimental model of adult mice”

**DOI:** 10.1111/jdi.14210

**Published:** 2024-04-13

Tohid Vahdatpour, Ali Nokhodchi, Parvin Zakeri‐Milani, Mehran Mesgari‐Abbasi, Naser Ahmadi‐Asl, Hadi Valizadeh Leucine–glycine and carnosine dipeptides prevent diabetes induced by multiple low‐doses of streptozotocin in an experimental model of adult mice. J Diabetes Investig. 2019; 10: 1177–1188. https://doi.org/10.1111/jdi.13018.

There is overlapping between the images in Figure 6 in the above article. The correct Figure 6 is shown below.

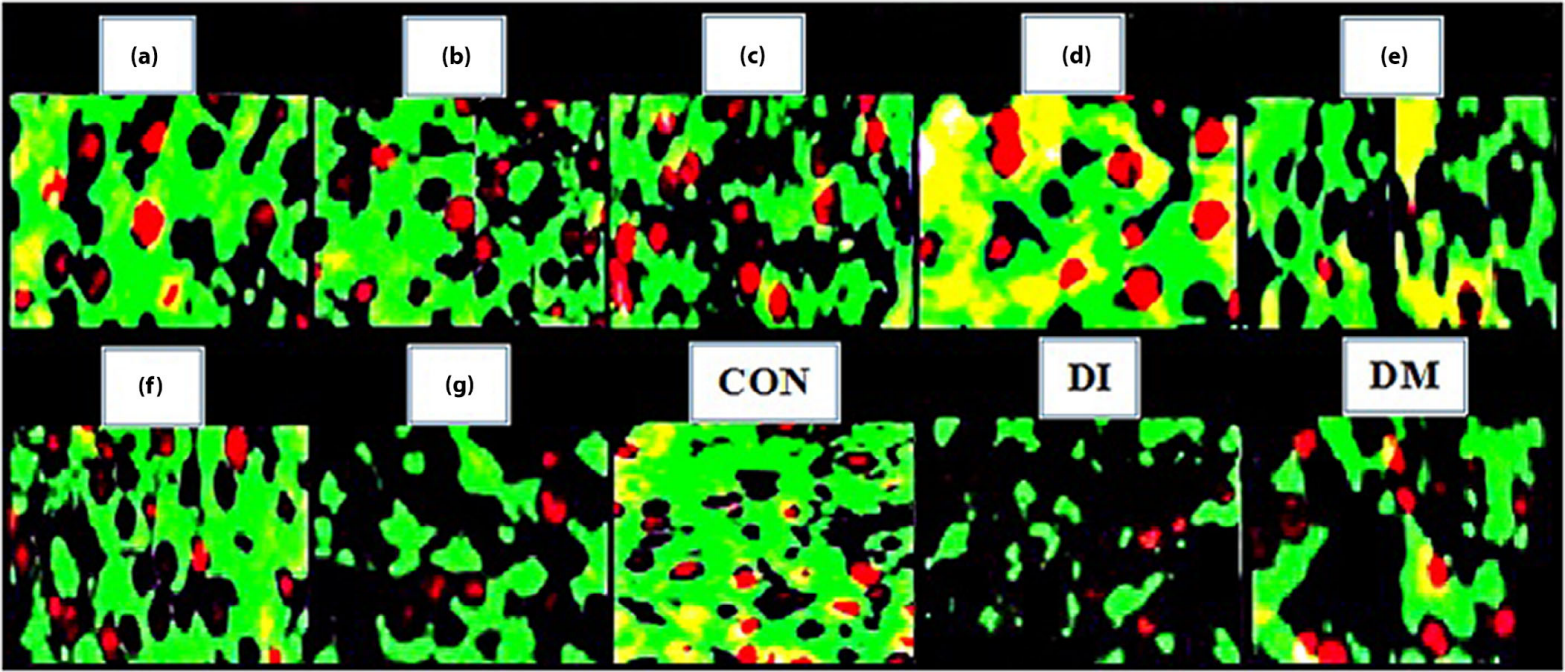

We apologize for the error.

